# Have Expandable Metallic Stents Replaced Operation for Malignant Biliary Obstruction?

**DOI:** 10.1155/1994/35089

**Published:** 1994

**Authors:** Prasit Watanapa, Robin C. N. Williamson

**Affiliations:** 1 Department of Surgery Siriraj Hospital, Mahidol University Thailand; 2 Department of Surgery Royal Postgraduate Medical School Hammersmith Hospital London W12 ONN UK


*HPB Surgery*, 1994, Vol. 8, pp. 151–157
Reprints available directly from the publisher
Photocopying permitted by license only

© 1994 Harwood Academic Publishers GmbH
Printed in Malaysia

### HPB INTERNATIONAL

**EDITORIAL & ABSTRACTING SERVICE**

JOHN TERBLANCHE, EDITOR
Department of Surgery,
Medical School · Observatory 7925
Cape Town · South Africa
Telephone: (021) 406-6232 Ext. 2
Telefax (021) 448-6461

### HAVE EXPANDABLE METALLIC STENTS REPLACED OPERATION FOR MALIGNANT BILIARY OBSTRUCTION?

### ABSTRACT

*Gordon, R. L., Ring, E. J., La Berge, J. M. and Doherty, M. M. (1992) Malignant biliary obstruction: Treatment with expandable metallic stents–Follow-up of 50 consecutive patients. Radiology, 182:697–701*

*Lillemoe, K. D., Sauter, P. K., Pitt, H. A., Yeo, C. J. and Cameron, J. L. (1993) Current status of surgical palliation of periampullary carcinoma. Surgery Gynecology and Obstetrics, 176:1–10*

**A consecutive series of 50 patients with malignant biliary obstruction were treated by means of palliative drownage with a metallic expandable stent. Stent placement was successful in all patients. The patients were followed up prospectively at 2-month intervals over a period of 9–22 months. Forty-one patients (82%) died; nine (18%) are still living. The overall patency and survival rates for the 50 patients were 5.8 months and 7.5 months, respectively. The 30-day mortality rate was 8% ($n = 4$), the minor complication rate was 18% ($n = 9$), and the major complication rate was 8% ($n = 4$). One patient (2%) had intrahepatic arterial bleeding that required embolization, one (2%) had a right subphrenic abscess, and two patients (4%) had transient septic events. Stent occlusion requiring a second intervention occurred in 24% of patients ($n = 12$). Excellent palliation was achieved in most patients. No stent migration occurred. No great clinical advantages in prolonged patency compared with those of other published series involving the use of plastic stents were demonstrated. Ease of placement and versatility may offset the high cost of the stent.**

**In recent years, the use of nonoperative palliation for unresectable periampullary carcinoma has increased markedly, in part, because of the high morbidity and mortality rates after surgical palliation. The current analysis was undertaken to determine whether or not decreases in morbidity and mortality rates, recently observed after resection of periampullary carcinoma, are now being seen in the surgical palliation of unresectable periampullary carcinoma. During a 54 month period, 118 consecutive patients underwent surgical exploration with the finding of unresectable periampullary adenocarcinoma. Jaundice was the most common complaint at admission, being present in 73**

**per cent of the patients. Abdominal or back pain, or both, was present in 71 per cent of the patients and weight loss was observed in 61 per cent of the patients. The most commonly performed procedure was combined biliary bypass and gastrojejunostomy, being performed upon 75 per cent of the patients. A gastroje-junostomy was performed upon 107 of 118 patients (91 per cent). The hospital mortality rate was 2.5 per cent. Postoperative complications occurred in 37 per cent of the patients but were seldom life-threatening. Wound infection was the most frequent postoperative complication (10 per cent), followed by cholangitis (8 per cent) and delayed gastric emptying (8 per cent). During the late follow-up period, only 4 per cent of the patients had gastric outlet obstruction, and only 2 per cent had recurrent jaundice. The mean survival time postoperatively was 7.7 months. These results demonstrate that patients with unresectable periampullary carcinoma can undergo surgical palliation with minimal perioperative mortality, acceptable morbidity and good long term palliation. We conclude that surgical palliation is the treatment of choice for carefully selected patients with unresectable periampullary carcinoma. *Surg. Gynecol. Obstet.*, 1993, 176:1–10.**

KEY WORDS: Malignant biliary obstruction carcinoma of the pancreas obstructive jaundice expandable metallic stents hepaticojejunostomy.

### PAPER DISCUSSION

During the 1980s percutaneous transhepatic stenting and endoscopic transpapillary stenting have become established methods for the palliation of jaundice in patients with malignant obstruction of the extrahepatic biliary tree. Several types of biliary endoprosthesis are now available, with large lumens ranging from 9 to 14 Fr in external diameter. Stent materials include Teflon® (E. I. du Pont, Wilmington, Delaware, USA), polyethylene and Percuflex® (MediTech, Watertown, Massachusetts, USA). Our review of articles published during 1983–1991 showed high success rates in terms of jaundice relief: between 76–100 per cent following percutaneous stent and 82–100 per cent following endoscopic stent. However, these types of endoprosthesis carry a high late complication rate, 28 per cent on average[1]. The major late complication leading to recurrent jaundice or cholangitis is clogging of the endoprosthesis, which occurs in 20–30 per cent of cases (whether placed from above or below). The self-expandable metal stent, Wallstent® (Medinvent SA, Lausanne, Switzerland) seems to solve the problem of clogging.

Davids and colleagues conducted a randomised trial of self-expandable stents (Wallstent®) versus polyethylene stents; both were placed endoscopically for distal malignant biliary obstruction[2]. The polyethylene stent used in this trial was a straight (10 Fr) endoprosthesis with two side flaps to prevent dislocation and a side hole at each end (PBN Medicals, Stenloese, Denmark). One hundred and five patients were included in this trial: 49 received a metal stent and 56 a straight polyethylene stent. The median period of patency was longer in the metal-stent group than in the polyethylene-stent group (273 days versus 126 days: $P = 0.006$). Stent occlusion occurred in 16 patients (33 per cent) in the metal-stent group after a median of 273 days compared with 30 patients (54 per cent) in the polyethylene-stent group after a median of 126 days. The main cause of metal-stent occlusion was tumour ingrowth through the meshes, whereas the main cause of polyethylene-stent occlusion was sludge encrustation.

Another report from the European Wallstent Study Group included 103 patients with malignant biliary obstruction from eight European endoscopy centres who underwent biliary decompression by means of Wallstent endoprosthesis. Although insertion of the stent was successful in nearly 100 per cent, long-term complications manifested by late cholangitis were seen in 18 patients after a median interval of 125 days. The cause of cholangitis was haemobilia in one case, distal tumour overgrowth in four, tumour ingrowth through the meshes of the stent in seven, biliary sludge obstruction in two, proximal bile duct compression in one and unknown in three.

It appears from these two studies that the occlusion rate by biliary sludge after metal stents is substantially less than that reported after polyethylene stents (21 per cent after a median time interval of 154 days[4]). However, the incidence of stent obstruction after metal stents is still high, being mostly caused by tumour ingrowth through the meshes of the stent or tumour overgrowth at either end. The paper by Dr. Gordon and colleagues shared these experiences. Fifty patients with malignant biliary obstruction were treated by a Wallstent: 24 patients had pancreatic carcinoma, 15 cholangiocarcinoma, 6 metastatic carcinoma from a variety of primary sites and 5 gallbladder carcinoma. Of these, nine patients (18 per cent) developed minor complications, four (8 per cent) major complications and four (8 per cent) died within 30 days of the procedure. Although stent placement was successful in all and early excellent palliation was achieved in most patients, twelve of fifty patients (24 per cent) had stent occlusion requiring one or more further interventions such as placement of additional stents ($n = 11$) or disimpaction of the sludge ($n = 1$). The causes of stent occlusion were tumour overgrowth in 10 cases, stent clogging by sludge and debris in one and uncertain in one. During the follow-up period of 9–22 months, there was no great improvement in patency for the Wallstent endoprosthesis compared with that for plastic stents reported by other studies. Due to the increasing use of this expandable stent during the past few years, more and more new complications have been reported (e.g. haemorrhage due to erosion of the stent through the duodenal wall[5] and acute pancreatitis[6]).

Surgical biliary-enteric bypass has been accepted for years as the procedure of choice to cope with obstructive jaundice due to irresectable malignant tumours. The report from Dr. Lillemoe and colleagues from the Johns Hopkins Medical Institutions confirms this statement. Among 118 patients with irresectable periampullary tumours who underwent palliative biliary bypass (89 cases or 75 per cent had combined biliary bypass and gastrojejunostomy), the hospital mortality rate was only 2.5 per cent and the 30-day mortality rate was 3.3 per cent. Recurrent jaundice occurred in only 2 per cent of those who survived the operation (two patients, at 6 and 12 months postoperatively). This experience was comparable to our own. Twenty-five patients with irresectable pancreatic head carcinoma underwent single-loop biliary and gastric bypass without a hospital death; two patients (8 per cent) had recurrent jaundice due to multiple liver metastases[7], but none developed renewed gastric outlet obstruction. By contrast, five patients in Lillemoe's series had gastric outlet obstruction at 3, 5, 5, 7 and 10 months postoperatively; in four of these five a gastrojejunostomy had been performed. These low figures for 30-day mortality and late complications (notably recurrent jaundice) in Lillemoe's series and our own compare well with those obtained by meta-analysis of 23 articles published between 1980–1990 (12 per cent deaths, 16 per cent late complications)[1].

During the past decade, there have been several studies reporting the outcome of nonoperative and operative treatment of irresectable periampullary tumours[1]. It can be concluded that operative and nonoperative techniques are equally effective in the short-term relief of jaundice, but operation offers a better long-term result. Many prospective randomised trials, particularly those published before 1990, showed that the surgical arm was associated with a higher mortality rate (20–24 per cent versus 8–20 per cent)[8–10]; these articles are usually quoted by authors who prefer nonoperative measures. There seems to have been a sharp drop in the mortality rate of palliative surgical bypass during the past few years[7,11,12] (and Lillemoe's paper), and this improvement reinforces our belief that an operative approach should be preferred in reasonably fit patients. Not only can laparotomy assess the resectability of the tumour more accurately than any imaging studies, but it can also palliate most symptoms at the same time (including pain) by the performance of biliary bypass, gastroenterostomy and chemical splanchnicectomy. Moreover, operation should forestall late complications, such as recurrent jaundice or duodenal obstruction. This benefit is important for any palliative procedure, since improvement in the quality of life is the principal aim of treatment.

We believe that the absence of late obstructive complications requiring readmission to hospital and the certainty that a resectable tumour is not overlooked are the main justifications for an operative approach to periampullary tumour. Expandable metallic stents have a role in patients with high surgical risk or those with very advanced tumour, whose life expectancy is less than a couple of months.

### REFERENCES

1. Watanapa, P. and Williamson, R. C. N. (1992) Surgical palliation for pancreatic cancer: developments during the past two decades. *Br. J. Surg.*, **79**, 8–20.
2. Davids, P. H., Groen, A. K., Rauws, E. A., Tytgat, G. N. J. and Huibregtse, K. (1992) Randomised trial of self-expanding metal stents versus polyethylene stents for distal malignant biliary obstruction. *Lancet*, **340**, 1488–1492.
3. Huibregtse, K., Carr-Locke, D. L., Cremer, M., Domschke, W., Fockens, P., Foerster, E., Hagenmüller, F., Hatfield, A. R. W., Lefebvre, J. F., Liquory, C. L., Matzen, P., Neuhaus, H., Sugai, B. M. and Williams, S. (1992) Biliary stent occlusion–a problem solved with self-expanding metal stents? *Endoscopy*, **24**, 391–394.
4. Huibregtse, K., Katon, R. M., Coene, P. P. and Tytgat, G. N. J. (1986) Endoscopic palliative treatment in pancreatic cancer. *Gastrointest. Endosc.*, **32**, 334–338.

5. Ee, H. and Laurence, B. H. (1992) Haemorrhage due to erosion of a metal biliary stent through the duodenal wall. *Endoscopy*, **24**, 431–432.
6. Van Steenbergen, W., Van Aken, L. and Ponette, E. (1992) Acute pancreatitis complicating the insertion of a self-expandable biliary metal stent. *Endoscopy*, **24**, 440–442.
7. Watanapa, P. and Williamson, R. C. N. (1993) Single-loop biliary and gastric bypass for irresectable pancreatic carcinoma. *Br. J. Surg.*, **80**, 237–239.
8. Bornman, P. C., Harries-Jones, E. P., Tobias, R., van Stiegmann, G. and Terblanche, J. (1986) Prospective controlled trial of transhepatic biliary endoprosthesis *versus* bypass surgery for incurable carcinoma of head of pancreas. *Lancet*, I: 69–71.
9. Shepard, H. A., Royle, G., Ross, A. P. R., Diba, A., Arthur, M. and Colin-Jones, D. (1988) Endoscopic biliary endoprosthesis in the palliation of malignant obstruction of the distal common bile duct: a randomised trial. *Br. J. Surg.*, **75**, 1166–1168.
10. Andersen, J. R., Sørensen, S. M., Kruse, A., Rollijaer, M., Matzen, P. (1989) Randomised trial of endoscopic endoprosthesis *versus* operative bypass in malignant obstructive jaundice. *Gut*, **30**, 1132–1135.
11. Singh, S. M., Longmire, W. P. Jr. and Reber, H. A. (1990) Surgical palliation for pancreatic cancer. The UCLA experience. *Ann. Surg.*, **212**, 132–139.
12. Kahn, P. J., Skornick, Y., Inbar, M., Kaplan, O., Chaichik, S. and Rozin, R. (1990) Surgical palliation combined with synchronous therapy in pancreatic carcinoma. *Eur. J. Surg. Oncol.*, **16**, 7–11.

Prasit Watanapa
Associate Professor of Surgery
Department of Surgery
Siriraj Hospital
Mahidol University
Thailand

Robin C. N. Williamson
Professor of Surgery
Department of Surgery
Royal Postgraduate Medical School
Hammersmith Hospital
London W12 ONN

### DOES PROPHYLACTIC ENDOSCOPIC SCLEROTHERAPY PREVENT VARICEAL BLEEDING OR NOT? A QUESTION OF EXPERIMENTAL DESIGN

Some circumstantial evidence is very strong,
as when you find a trout in the milk Thoreau

### ABSTRACT

*Van Thiel, D. H., Dindzans, V. J., Schade, R. R., Rabinovitz, M. and Gavaler, J. S. (1993) Prophylactic versus emergency sclerotherapy of large esophageal varices prior to liver transplantation. Digestive Diseases and Sciences; 38 1505–1510*

**From January 1985 through July 1987, adult patients accepted for liver transplantation with large esophageal varices were enrolled in a study evaluating the use of prophylactic vs emergency sclerotherapy. Six hundred forty-eight subjects received prophylactic sclerotherapy, and 172 received emergent sclerotherapy. Esophageal stricture formation was increased 12.9-fold ($P < 0.001$), esophageal perforation 6.4-fold ($P < 0.005$), and post-sclerotherapy bleeding esophageal ulcers 3.7-fold ($P < 0.001$) in those receiving emergency sclerotherapy as opposed to prophylactic sclerotherapy. These differences were even greater if the number of sclerotherapy sessions rather than the number of patients was used as the denominator for the comparisons. In total, 19.6% of emergency sclerotherapy cases were associated with an untoward outcome of sclerotherapy; only 1.9% of cases receiving prophylactic sclerotherapy experienced an untoward outcome ($P < 0.001$). These data demonstrate that emergency sclerotherapy is associated with a greater prevalence of complications and support earlier studies that show that sclerotherapy**

